# Large waves and navigation hazards of the Eastern Mediterranean Sea

**DOI:** 10.1038/s41598-022-20355-9

**Published:** 2022-10-03

**Authors:** Sagi Knobler, Dan Liberzon, Francesco Fedele

**Affiliations:** 1 School of Civil & Environmental Engineering, Georgia Institute of Technology, Atlanta, GA 30332 USA; 2 Interdisciplinary Program for Ocean Engineering, The Technion-Israel Institute of Technology, 320003 Haifa, Israel; 3 Faculty of Civil and Environmental Engineering, The Technion-Israel Institute of Technology, 320003 Haifa, Israel; 4 CAMERI-Coastal and Marine Engineering Research Institute, 320003 Haifa, Israel

**Keywords:** Natural hazards, Physical oceanography, Statistics

## Abstract

We present a statistical analysis of deep-water buoy measurements of large waves generated during two major storms of the Eastern Mediterranean in 2017 and 2018, respectively. The largest waves observed do display similar characteristics to those of the Draupner, Andrea, and El Faro rogue waves in that second order bound nonlinearities enhance the linear dispersive focusing of extreme waves. We also present a novel analysis of waves in space-time to predict potential risks posed by such large waves to navigation. In particular, we consider the scenario of two types of vessels of the Israeli Navy fleet navigating during the most intense stages of the two storms considered here and provide predictions for the largest waves likely to be encountered.

## Introduction

In light of global warming, weather events are likely to become more severe and hazardous to coastal and offshore structures and maritime navigation. Not only does this relate to vast oceans, but also to large basins such as the Black Sea and the Mediterranean Sea. In particular, the Eastern Mediterranean deserves special attention because it lacks studies on the physical characteristics of extreme sea states and large waves. A climatology hot-spot and natural treasures area, the Eastern Mediterranean is a basin of increasing interest, both ecologically and economically^1^. Thus, a scientific investigation and prediction of wave extremes are of paramount importance, especially for rogue wave predictions and maritime navigation in extreme weather.

Conventional thresholds^2^ have been adopted to distinguish if a wave is rogue or not. In particular, a wave is rogue if its crest height $h>1.25 H_s$  or the crest-to-trough (wave) height $H>2 H_s$, where $H_s$ is the significant wave height of the sea state in which the rogue wave occurred. For example, the first famous rogue wave ever recorded is the Draupner wave that occurred on the $1{st}$ of January 1995 in an intense sea state with $H_s=11.9$ m^3, 4^. The 26-m rogue wave hit the lower deck of the “Draupner S” oil platform at the North Sea. The Draupner wave has $H=2.15H_s$ and $h=1.55H_s$ satisfying the conventional criteria for being rogue^2^. On November 2007, the powerful Andrea storm crossed the “Ekofisk” oil platform. In a sea state with $H_s=9.2$ m four wave gauge lasers mounted on the rig measured the Andrea rogue wave^5^, whose wave height $H=2.3H_s$ and crest height $h=1.62H_s$. In November 2020 a rogue wave has been detected off the Vancouver Island in the Atlantic Ocean^6^, whose wave height $H=2.92H_s$ and crest height $h=1.98H_s$ are well over the conventional rogue thresholds^2^. Nevertheless, the crest height of that rogue wave reached 11.96 m in amplitude, and thus smaller than both the Draupner and Andrea waves.

There is a lack of scientific knowledge about the metocean characteristics of the eastern part of the Mediterranean Sea, especially for rogue wave predictions and vessel navigation in extreme weather. Reports of rogue wave occurrences involving vessel navigation have been mostly observed in the western part of the Mediterranean by eyewitnesses on cruise ships that have been impacted by extreme waves resulting in structural damage and loss of lives. Two cases have been recently reported: the “Voyager” accident that occurred in 2005 and the “Louis Majesty” accident in 2010. Both accidents triggered scientific interest to confirm, or rebut the possibility of cruise ships encountering rogue waves^7, 8^. A rogue wave event has been caught in the Northern Adriatic Sea by a web-camera placed on the oceanographic tower ’Acqua Alta’ off the Venice coast, Italy. The estimated wave crest height is in the range of 5.1–6.4 m^9^ and exceeded the conventional rogue thresholds^2^. All the above-mentioned extreme waves have been observed in the western part of the Mediterranean Sea.

Recently, Knobler et al.^10^ provided the first observations of several rogue wave events in the Eastern part of the Mediterranean Sea from measurements acquired by AXYS Technologies “3 metre” metadata buoy in deep waters (see “Methods” section for details). Surface elevations, wind and wave parameters were also collected by a meteorological station^10, 11^. As expected for this region, the strongest storm events occurred throughout October–April period^10^. They detected a sample of 109 rogue waves, whose wave heights exceeded the rogue threshold $2 H_s$, and only nine waves out of the sample had crests exceeding $1.25 H_s$ in height^2^. However, no wave crest exceeded 4 m in height.

In the present work, we further examine the statistics of large waves in deep waters observed during the two most powerful storm events of the Eastern Mediterranean recorded during the last five years. In particular, the first storm occurred during January 16–22 in 2018, and hereafter referred to as Gaia. The second storm occurred during January 25–31 in 2017, and hereafter referred to as Jan 2017. We note that the largest waves observed in both storms do not exceed the rogue wave thresholds^2^, in contrast to the Draupner and Andrea rogue waves^3–5, 12, 13^ that occurred in intermediate water depths and did reach crest heights of $1.6H_s$ well over the rogue threshold. In our studies, we also exploit statistical distributions for non-stationary time series^14^ to analyze the statistics of surface elevations sampled from the two storms. Finally, we carry out a novel space-time analysis of the most intense sea states as a basis for predicting potential rogue hazards for ships navigating through the rough waters of the storms.

## Results

This section is structured as follows. First, we discuss the metocean characteristics of the sea states generated by the two observed storms as they passed through the Eastern Mediterranean. Relevant wave parameters and statistical models are defined in the “Methods” section. Then, we make prediction on the extreme waves during the two storms. To do so, we draw on Fedele et al.^14^ and use their formulation of the probability structure of non-stationary time series as a model for the sequence of sea states in a storm. In particular, we study the occurrence frequencies of rogue waves during Jan 2017 and Gaia storms encountered by an observer at a given point of the sea surface. The wave extremes observed at the peak of the storms and their characteristics are then compared to those of the Andrea and Draupner rogue waves, observed at different oil platforms in the North Sea in 1995 and 2007, respectively^12^ as well as the simulated El Faro rogue wave^13^. The metocean parameters of these five sea states are summarized in Table 1. Finally, we present a space-time wave analysis^13, 15, 16^ of the most intense sea states to predict rogue hazards for vessel navigation within an area.

**Table 1:** Wave parameters and various statistics of Jan 2017 and Gaia at the storm peak in comparison to the El Faro, Andrea and Draupner rogue sea states^12^.

	El Faro	Andrea	Draupner	Jan 2017	Gaia
Significant wave height $H_{s}$ [m]	9.0	10.0	11.2	6.86	6.16
Dominant wave period $T_{p}$ [s]	10.2	14.3	15.0	11.9	14.1
Mean zero-crossing wave period $T_{0}$ [s]	9.2	11.6	11.9	8.6	9.2
Mean wavelength $L_{0}$ [m]	131	209	219	115	131
Depth *d* [m], $k_0 d$ with $k_0=2\pi /L_0$	4700, 2.25	74, 2.23	70, 2.01	258,14.11	258, 12.36
Spectral bandwidth $\nu$	0.49	0.35	0.36	0.43	0.46
Angular spreading $\sigma _{\theta }~[rad]$	0.79	0.37	0.39	1.08	1.11
Parameter $R=\sigma _{\theta }^{2}/2\nu ^{2}$	1.34	0.56	0.59	3.18	2.88
Benjamin Feir Index *BFI* in deep water^17^	0.36	0.24	0.23	0.16	0.09
Tayfun NB skewness $\lambda _{3,NB}$^18^	0.262	0.159	0.165	0.222	0.199
Observed skewness $\lambda _{3}$	0.162	0.141	0.146	0.103	0.105
Maximum NB dynamic excess kurtosis $\lambda _{40,\textit{max}}^d$^19^	$10^{-3}$	$2.3 \times 10^{-3}$	$2.1 \times 10^{-3}$	$-4.8 \times 10^{-3}$	$-1.6 \times 10^{-3}$
Janssen NB bound excess kurtosis $\lambda _{40,NB}^{d}$ ^20^	0.049	0.065	0.074	0.097	0.087
Observed excess kurtosis $\lambda _{40}$	0.042	0.041	0.032	0.100	0.087
Actual maximum crest height $h/H_{s}$	1.68	1.63	1.55	1.04	1.11
Actual maximum crest-to-trough (wave) height $H/H_{s}$	2.6	2.30	2.10	1.98	1.96

We refer to the “Methods” section for the definitions of wave parameters.

Our extreme wave analysis focused on the study of the time sequence of changing sea states during the two storms. While the analysis of Fedele et al.^14^ yields an optimal sea state duration $T_{sea}$ of 40–50 min to minimize the variation between waves of consecutive sea states of storms, the duration *T* of our actual measurements was limited to 18 min at the beginning of each hour to minimize data storage. So, the analyses here were unavoidably based on $T_{sea}=T=18$ min. Nevertheless, we observed that our wave statistics are very robust to changes in $T_{sea}$ up to $\pm 5$ min, provided that surface elevations of each sea state in the storm sequence are normalized by the respective significant wave height.

### Metocean parameters

**Figure 1:**
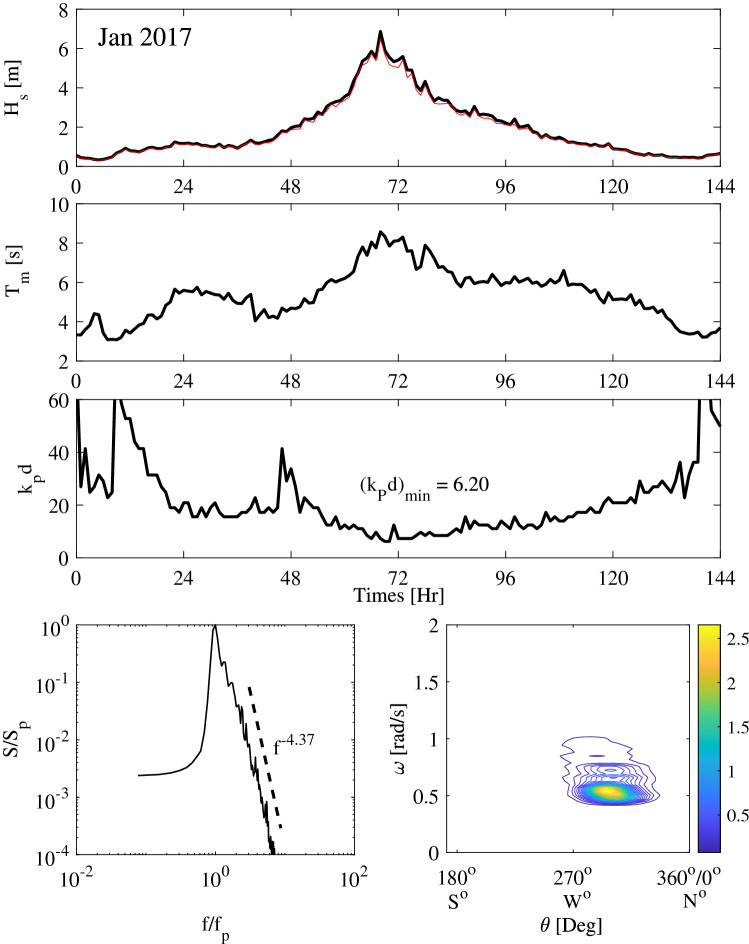
Metocean parameters history during January 2017 storm: (top panels) hourly variation of the significant wave height estimated as $H_s=4\sigma$ (black) and $H_{1/3}$ (red), mean zero-up-crossing wave period $T_0$, and depth factor $k_p d$; (bottom-left) normalized frequency spectrum $S(f)/S_p$ and (bottom-right) the directional spectrum $S(\omega ,\theta )$ ($[\mathrm {m}^2/\text {rad/s/deg}]$) at the storm peak. Here, $S_p$ ($[\mathrm {m}^2/\text {Hz}]$) and $f_p$ ($[\mathrm {Hz}]$) are the spectral amplitude and frequency at the storm peak.

**Figure 2:**
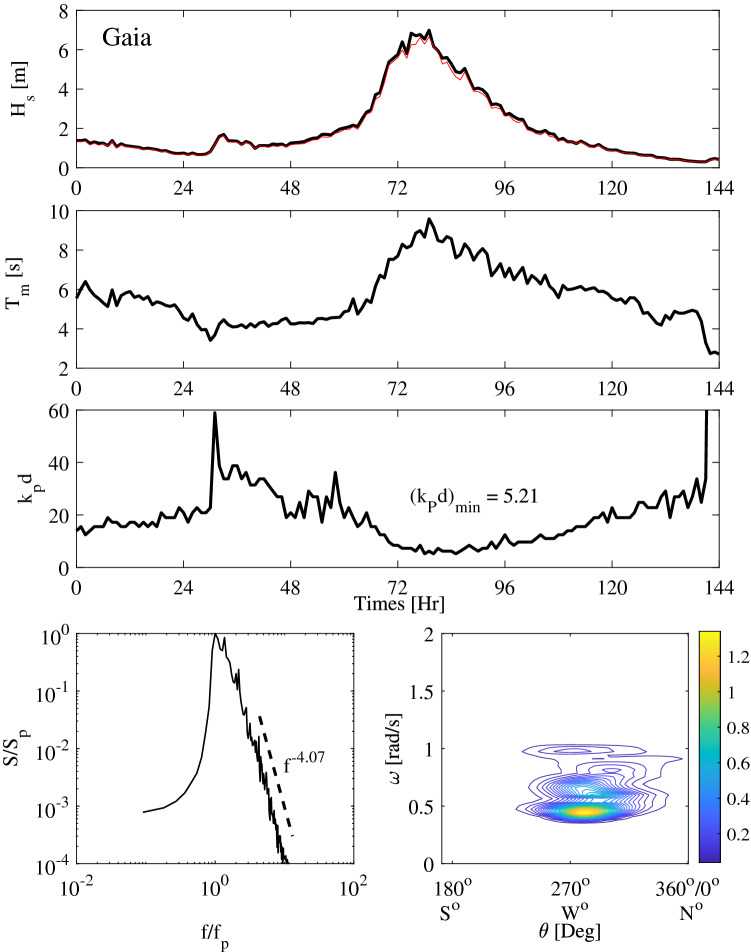
Metocean parameters history during Gaia storm: (top panels) hourly variation of the significant wave height estimated as $H_s=4\sigma$ (black) and $H_{1/3}$ (red), mean zero-up-crossing wave period $T_0$, and depth factor $k_p d$; (bottom-left) normalized frequency spectrum $S(f)/S_p$ and (bottom-right) the directional spectrum $S(\omega ,\theta )$ ($[\mathrm {m}^2/\text {rad/s/deg}]$) at the storm peak. Here, $S_p$ ($[\mathrm {m}^2/\text {Hz}]$) and $f_p$ ($[\mathrm {Hz}]$) are the spectral amplitude and frequency at the storm peak.

**Figure 3:**
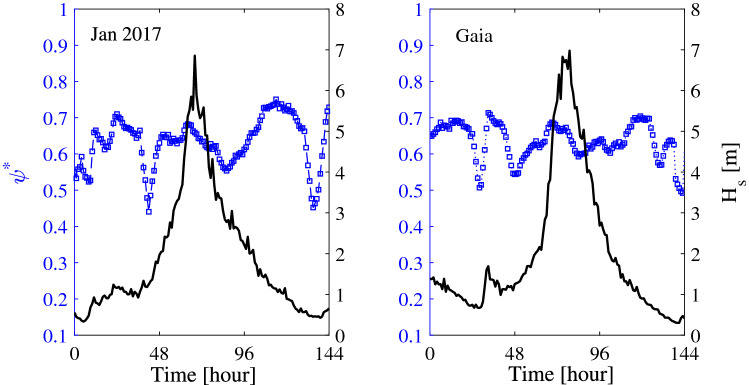
Metocean parameters history during storms  Jan 2017 (left panel, start date Jan 25, 2017, 00:00) and Gaia (right panel, start date Jan 16, 2017, 00:00): hourly variation of Boccotti parameter $\psi ^*$ and the significant wave height estimated as $H_s=4\sigma$.

The metocean parameters history of Jan 2017 storm are shown in Fig. 1. In particular, the top panel depicts the hourly variation of the significant wave height. This is estimated as $H_{s}=4\sigma$ (black line). For comparison, $H_{1/3}$ is also shown (red line), which is $5\%$ smaller than $H_{s}$. The mean zero-up-crossing wave period $T_{0}$ is shown in the second panel of the same figure, and the hourly variations of $k_{p} d$ are reported in the third panel, where $k_p$ is the peak wavenumber. The bottom panel on the left depicts the frequency spectrum measured at the storm peak. The directional wave spectrum is also shown, in the bottom panel on the right. Similarly, the histories of metocean parameters of storm Gaia are presented in Fig. 2. The two storms generated waves in deep waters depth ($k_p d>\pi$, see^14^). Moreover, $k_{p}d$ is well above the critical threshold 1.363, at which modulation instability disappears. Nevertheless, third-order nonlinear modulational effects are negligible because the energy of the two sea states spreads both in all directions and frequencies^12, 19^ as clearly seen in the bottom panels of Figs. 1, 2. Furthermore, we depict in Fig. 3 the Boccotti parameter $\psi ^*$, which measures the spectral bandwidth of the sea states of the two storms. The values of the observed $\psi ^*$ vary in the range [0.5–0.7] indicating sea states with broadband spectra. In Table 1 we report the metocean parameters of the two storm events at their peak stages in comparison to those of the El Faro, Draupner and Andrea rogue sea states^12, 13^. Note that the five sea states all have similar metocean characteristics. Both Jan 2017 and Gaia are in deep waters and developed comparable steep sea states as the other three sea states, even if their peak significant wave height is approximately $30\%$ smaller than that of the other rogue sea states. Indeed, the observed wave parameters have comparable values in the same range typical of oceanic sea states in storms. Our analysis indicates that the largest observed waves in Gaia and Jan 2017 storms have characteristics quite similar to those displayed by the El Faro, Andrea and Draupner rogue waves^12, 13^, where second order bound nonlinearities are the dominant factor that enhances the linear dispersive focusing of extreme waves.

### Statistical measures of ocean nonlinearities

We use integral statistics such as the Tayfun steepness $\mu =\lambda _3/3$ and the excess kurtosis $\lambda _{40}$ of the zero-mean surface elevation $\eta (t)$ to measure ocean wave nonlinearities. Wave skewness $\lambda _3$ describes the effects of second-order bound nonlinear effects, which alter the geometry and statistics of the sea surface with higher sharper crests and shallower more rounded troughs^23–25^. The excess kurtosis indicates deviations of the distribution of surface elevations from Gaussianity. It comprises a dynamic component^19, 26^ $\lambda _{40}^{d}$ measuring third-order quasi-resonant wave-wave interactions and a bound contribution $\lambda _{40}^{b}$ induced by both second- and third-order bound nonlinearities^23–28^.

**Figure 4:**
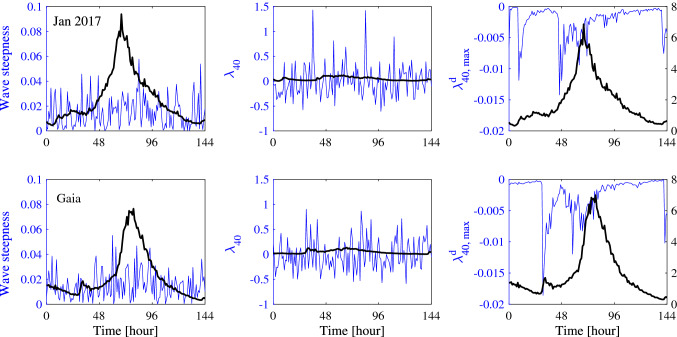
Statistical parameters history during storms (top panels) Jan 2017 and (bottom panels) Gaia: (left) hourly variation of the theoretical NB (bold line) and observed Tayfun steepness $\mu =\lambda _3/3$ (thin blue line), (center) observed excess kurtosis (thin blue line) and NB estimate (bold line) and (right) theoretical maximum NB dynamic excess kurtosis^19^ (blue thin line). The significant wave height history is also reported (black line).

Figure 4 depicts the statistical parameters history for the two analyzed storms. The top panels display the hourly variations of the observed Tayfun steepness $\mu =\lambda _3/3$, the observed excess kurtosis $\lambda _{40}$ and the theoretical maximum Narrow Band (NB) dynamic excess kurtosis^19^ $\lambda _{40,max}^d$ for storm Jan 2017. The theoretical narrow-band predictions are also shown. The same comparisons for storm Gaia are also reported in the bottom panels. In Table 1 we compare the statistical parameters of Gaia and Jan 2017 strongest sea states and the Draupner and Andrea rogue sea states (from Fedele et al.^14^). All sea states are short-crested as characterized by large directional spreading, especially at the peak of the storms as $R>1$. In open ocean, wave energy can spread directionally and third-order quasi-resonant nonlinearities are essentially insignificant^12, 19, 29–32^ in such realistic oceanic conditions^12, 33, 34^. Indeed, the right panels of Fig. 4 indicate that the maximum dynamic excess kurtosis is of $O(10^{-3})$ and negative for both Jan 2017 and Gaia, and negligible in comparison to the associated bound component. The values of the observed excess kurtosis from the hourly 18 min samples are erratic due to the short record duration. Estimates of the third order moment, or wave skewness from the buoy’s time records of surface elevations converged for time series 70–100 wave periods long, or 12–15 min records. In contrast, estimates of the fourth-order moment, or excess kurtosis were at the verge of convergence for the available 18-min record. Nevertheless, our analysis indicates that third-order quasi-resonant interactions, including NLS-type modulational instabilities play an insignificant role in the formation of large waves^12, 19^ in agreement with oceanic observations available so far^24, 35, 36^.

**Figure 5:**
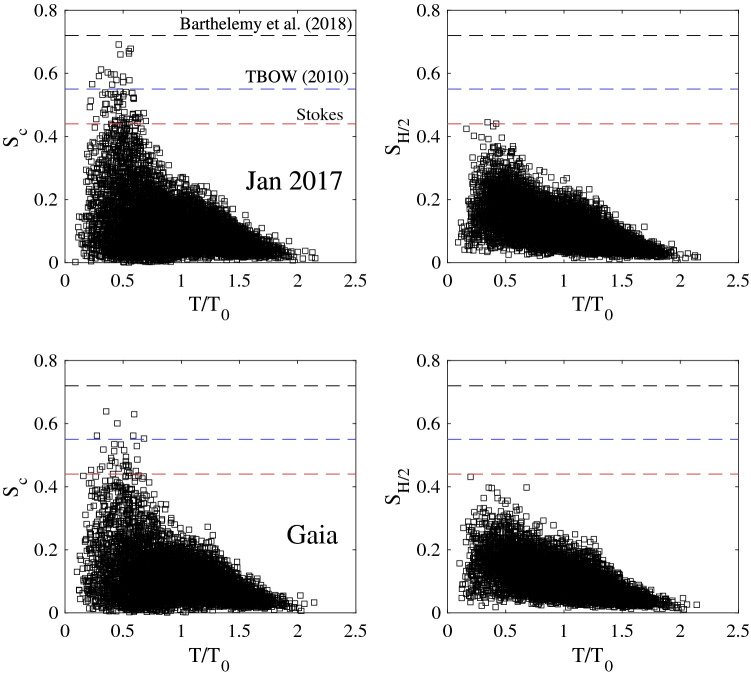
Observed Local steepnesses $S_c=k h$ and $S_{H/2}=kH/2$ versus local period $T/T_0$ for (top panels) Jan 2017 and (bottom panels) Gaia storms. Stokes limit = 0.448, TWBO limit^21^ = 0.55 and Barthelemy et al.^22^ = 0.72. *k* = local wave number, *h* = crest height, *H* = crest-to-trough height and $T_0$ = mean zero-crossing period. Because of the crest-trough asymmetry, on average $h>H/2$ and $S_c>S_{H/2}$.

Next we analyze the local steepness of the individual waves observed. To do so, we consider the two definitions $S_c=k h$ and $S_{H/2}=k H/2$ based on the crest height *h* and the half of the crest-to-trough height *H*, respectively. Here, *k* is the wavenumber estimated from the measured local wave period using the nonlinear dispersion relation^21, 37^. Toffoli et al.^21^ used $S_{H/2}$ to analyze the statistics of individual wave steepnesses from both laboratory and open sea measurements. They concluded that waves can reach $S_{H/2}$ values above the Stokes limit 0.448 and up to the limit 0.55, hereafter referred to as TBOW. Clearly, *H*/2 is smaller than the crest height *h* on average because of the crest-trough asymmetry due to bound nonlinearities^23^. As a consequence $S_{H/2}$ is smaller than $S_c$. Indeed, in a numerical study of breaking wave groups Barthelemy et al.^22^ observed $S_c=k h$ values that did not exceed the limit 0.72, which is based on the fifth order Stokes wave solution^38^. Recently, Knobler et al.^39^ observed in their experiments waves of average amplitude with steepness $S_c$ above 0.8. The steepest waves appear as forms of wave dislocations due to crest pairing, or splitting^40^. However, the largest waves in their dataset did not exceed the theoretical limit^22^ 0.72. Figure 5 depicts the local steepness of the individual waves measured during Jan 2017 and Gaia storms. Note that the observed $S_{H/2}$ values do not exceed the TBOW limit 0.55. Similarly, the observed $S_{c}$ values do not exceed the 0.72 limit proposed by Barthelemy et al.^22^.

### Occurrence frequency of a rogue wave during storms

We now describe the statistics of large waves encountered by an observer at a fixed point of the ocean surface during a storm of duration $T_s$. To do so, we draw on Fedele et al.^14^ and model the storm as a non-stationary continuous sequence of sea states of duration *dt*, and $dt/T_0(t)$ is the number of waves in the sea state, where $T_0(t)$ is the time-dependent mean zero-crossing wave period. We want to estimate the probability, or occurrence frequency $P_{ns}(\xi )$ of a wave crest height exceeding a given threshold $\xi H_s$ as encountered by a fixed observer, where $H_s=4\sigma$. That is the probability to randomly pick from the non-stationary time series observed at a fixed point a wave crest that exceeds the threshold $\xi H_s$, and it is given by^14^

1 $$\begin{aligned} P_{ns}(\xi )=\frac{\int _{0}^{T_{s}}P(\xi =h/H_{s}(t),t) \frac{1}{T_{0}(t)}dt}{\int _{0}^{T_{s}}\frac{1}{T_{0}(t)}dt} , \end{aligned}$$

where $P(\xi ,t)$ is the stationary exceeding probability of the sea state occurring in the time interval *t* and $t+dt$, which depends on wave parameters around time *t*. The definition of $P_{ns}$ is consistent with the way wave crests are sampled from non-stationary wave measurements during storms. Moreover, the threshold $\xi H_s$ is exceeded on average once every $N_{h}(\xi )=1/P_{ns}(\xi )$ waves, where $N_{h}$ is also the conditional return period of a wave whose crest height exceeds $\xi H_s$. In practice, the non-stationary $P_{ns}$ is estimated as the weighted average^14^

2 $$\begin{aligned} P_{ns}(\xi )=\frac{\sum _{j=1}^{N_{s}}P(\xi =h/H_{s}(t_{j}))N_{w,j}}{\sum _{j=1}^{N_{s}}N_{w,j}}, \end{aligned}$$

where $N_{w,j}$ is the number of waves sampled in the sea state *j* and a storm is partitioned in a finite sequence of $N_s$ sea states of duration $D_s$. For weakly nonlinear random sea states (stationary seas), the probability *P* is hereafter described by the third order Tayfun-Fedele^24^ (TF), modified narrow-band^41^ (MNB), Tayfun^23, 25^ (T), Forristall^43^ (F) and the linear Rayleigh (R) distributions (see “Methods” section). Similarly, the non-stationary occurrence frequency $P_{ns}(H)$ of a wave in a storm whose crest-to-trough (wave) height exceeds the threshold $H/H_s$ are described by the same Eqs. (1) by simply replacing *P*(*h*) with the exceedance probability *P*(*H*) proper for wave heights of stationary seas. This is hereafter described by the generalized Boccotti^42^ (B), Tayfun^44^ (T) and linear Rayleigh (R) distributions (see “Methods” section).

**Figure 6:**
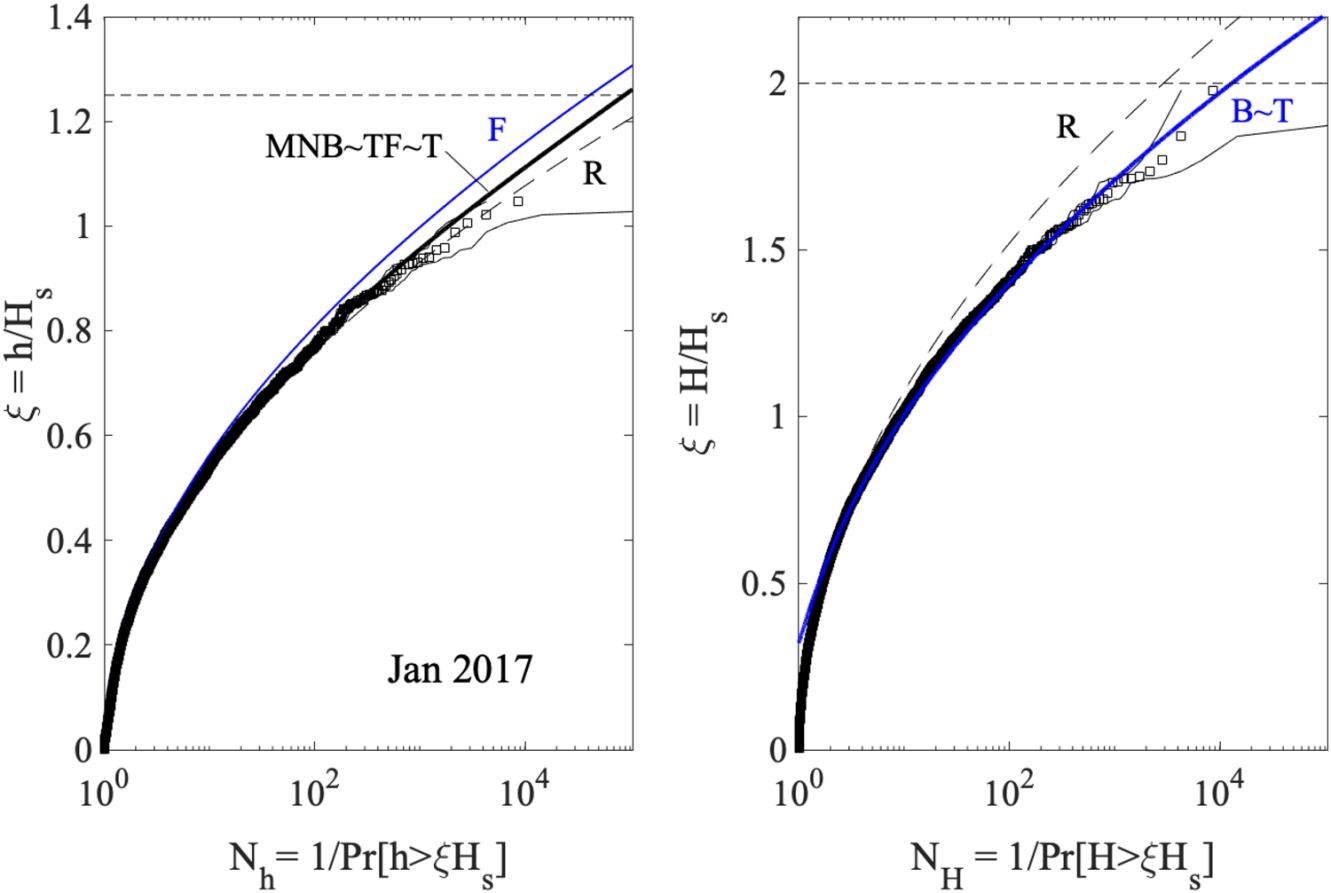
Wave statistics for storm Jan 2017: (left) crest heights $h/H_s$ and (right) crest-to-trough wave heights $H/H_s$ versus number of waves $N_h(\xi )$. The empirical distribution (squares) from the observed wave ensemble is compared against with theoretical models: T = Tayfun^23, 25^, TF = Tayfun-Fedele^24^, MNB = modified narrow-band^41^, B = generalized Boccotti^42^, F = Forristall^43^ and R = Rayleigh distributions. Confidence bands are also shown (light dashes). $N_h(\xi )$ is the inverse of the exceedance probability $\mathrm {Pr}[h>\xi H_s]$, and similarly $N_H(\xi )$ is for crest-to-trough heights. Horizontal lines denote the rogue threshold $1.25H_s$ and $2H_s$ for crest and wave heights^2^.

**Figure 7:**
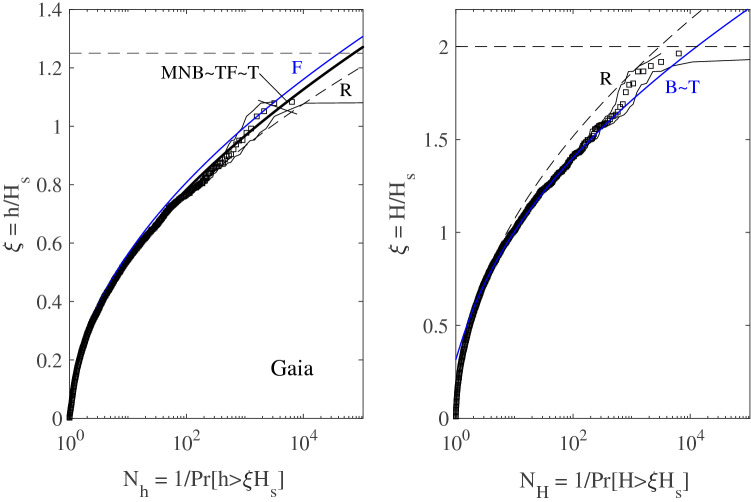
Wave statistics for storm Gaia: (left) crest heights $h/H_s$ and (right) crest-to-trough wave heights $H/H_s$ versus number of waves $N_h(\xi )$. The empirical distribution (squares) from the observed wave ensemble is compared against with theoretical models: T = Tayfun^23, 25^, TF = Tayfun-Fedele^24^, MNB = modified narrow-band^41^, B = generalized Boccotti^42^, F = Forristall^43^ and R = Rayleigh distributions. Confidence bands are also shown (light dashes). $N_{h}(\xi )$ is the inverse of the exceedance probability $\mathrm {Pr}[h>\xi H_s]$ for crest heights, and similarly $N_H(\xi )$ is for crest-to-trough heights. Horizontal lines denote the rogue threshold $1.25H_s$ and $2H_s$ for crest and wave heights^2^.

Figure 6 summarizes the wave statistics for storm Jan 2017. In particular, the left panel depicts the empirical distribution (squares) for crest heights $h/H_s$ plotted versus the number of waves $N_h(\xi )$. This is compared against theoretical predictions of the non-stationary second-order F, MNB and T models as well as the third-order TF and Rayleigh (R) distributions. T and TF models are based on the observed Tayfun steepness $\mu =\lambda _3/3$. Note that TF is practically the same as MNB as an indication that second-order effects are dominant. F slightly overestimates the return periods, whereas the linear R model underestimates them. This indicates the dominance of second-order nonlinearities in shaping the sea surface. Similarly, the right panel of the same Fig. 6 reports the empirical distribution for crest-to-trough wave heights for Jan 2017. The observed statistics is well described by both the generalized Boccotti (B) and Tayfun (T) models. Similar conclusions also hold for the wave statistics for Gaia presented in Fig. 7.

**Figure 8:**
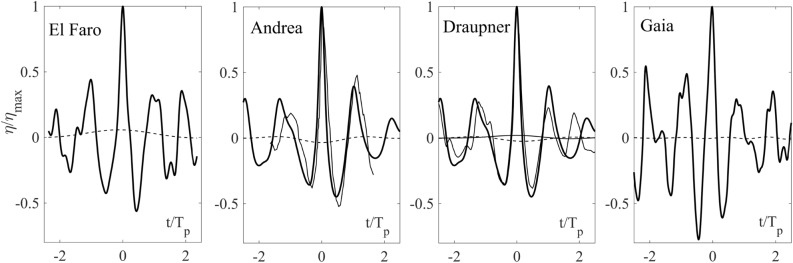
Simulated wave profiles $\eta /\eta _{max}$ (solid) and mean sea levels (MSL) (dashed) versus $t/T_p$ for (from left to right) El Faro, Andrea and Draupner sea states. Gaia largest wave observed is shown in the right panel. Actual measurements (thin solid) and MSLs (thin solid) are also shown for Draupner. Note that the Gaia MSL is insignificant and the Andrea MSL is not available. $\eta _{max}$ is the maximum crest height and $T_p$ is the dominant wave period (see Table 1 and “Methods” section for definitions).

The wave profile $\eta$ with the largest wave crest height observed during Gaia ($h=1.11 H_s\approx 6.8$ m, $H_s=6.16$ m) is shown in the right panel of Fig. 8. For comparison, the El Faro, Draupner and Andrea rogue wave profiles are also shown^12, 13^. From Table 1, Gaia wave did not exceed the rogue threshold $1.25 H_s$ as the other three rogue waves did. Nevertheless, the five sea states have very similar characteristics, suggesting a common generation mechanism due to linear dispersive focusing enhanced by second-order nonlinearities^12^.

### Wave statistics in space-time

The maximum surface wave height observed over a given area during a time interval, or space-time extreme is much larger than the maximum observed at a given point over the same time period^15, 16, 48^. Indeed, in the short-crested directional seas typically generated by storms it is very unlikely that an observed large crest at a given point in time is actually the largest crest of a group of waves propagating in space-time. In contrast, it is most likely that the sea surface was much higher somewhere near the measurement point, as Boccotti’s (2000) QD theory predicts.

Euler Characteristics of random fields^49–51^ provide a theoretical framework for modelling space-time wave extremes stochastically^15, 16, 52^. Drawing on Fedele et al.^13^ consider a 3-D non-Gaussian field $\eta (x,y,t)$ in space-time over an area *A* for a time period of *D*. The area cannot be too large since the wave field may not be statistically homogeneous. The duration should be short so that the sea state can be assumed as stationary and spectral changes insignificant. Then, drawing on the linear Gaussian space-time model^49–51^ the third-order nonlinear probability $P_{\mathrm {ST}}(\xi ;A,D)$ that the maximum surface elevation $\eta _{\max }$ over the area *A* and during the time interval *D* exceeds the generic threshold $\xi H_{s}$ is described by^13, 49–51^

3 $$\begin{aligned} P_{\mathrm {ST}}(\xi ;A,D)=\mathrm {Pr}\left\{ \eta _{\max }>\xi H_{s}\right\} =(16M_3\xi _{0}^{2}+4M_2\xi _{0}+M_1)P_{\mathrm {TF}}(\xi ), \end{aligned}$$

where

4 $$\begin{aligned} P_{\mathrm {TF}}(\xi )=\exp \left( -8\xi _{0}^{2}\right) \left[ 1+ \varLambda \xi _{0}^{2}(4\xi _{0}^{2}-1)\right] , \end{aligned}$$

denotes the third-order nonlinear Tayfun-Fedele model^24^ for the probability of exceedance of crest heights at a fixed point. The parameter $\varLambda$ measures third-order nonlinear effects and can be estimated from the excess kurtosis as $\Lambda_{appr}={8\lambda _{40}}/{3}$ (see “Methods” section). Note that our analysis indicates that third-order nonlinearities are negligible. The amplitude $\xi$ relates to the Rayleigh-distributed envelope $\xi _0$ via the Tayfun quadratic equation^11, 23, 25^

5 $$\begin{aligned} \xi =\xi _{0}+2\mu \xi _{0}^{2}. \end{aligned}$$

The space-time parameters $M_{1}$ and $M_{2}$ are the average number of 1-D and 2-D waves that can occur on the edges and boundaries of the volume $\Omega$, and $M_{3}$ is the average number of 3-D waves that can occur within the volume^15^. These all depend on the directional wave spectrum and its spectral moments defined in the “Methods” section.

Given the probability structure of the linear surface wave maximum in Eq. (3), the nonlinear mean maximum surface or crest height $\overline{h}_{\mathrm {ST}}=\xi _{\mathrm {ST}}H_s$ attained over the area *A* during a time interval *D* is given, according to Gumbel (1958), by^13^

6 $$\begin{aligned} \xi _{\mathrm {ST}}=\overline{h}_{\mathrm {ST}}/H_{s}= \xi _{\mathrm {m}}+\gamma _{e}\beta , \end{aligned}$$

where $\beta =1/P'_{ST}(\xi )$ is the inverse of the derivative of $P_{ST}(\xi )$ with respect $\xi$, the Euler-Mascheroni constant $\gamma _{e}\approx ~0.577$, and the most probable surface elevation value $\xi _{\mathrm {m}}$ follows from Eq. (3) as

7 $$\begin{aligned} P_{\mathrm {ST}}(\xi _{\mathrm {m}};A,D)=1. \end{aligned}$$

Fedele et al.^13^ observed that Gaussian space-time models^53–57^ overestimate the maximum surface height over large areas and time intervals because they rely on Gaussianity. Indeed, Gaussian seas do not account for the wave physics of nonlinear dispersion^29^, which limits the wave growth as a precursor to breaking^29, 58^. As a result, water surface elevations of a Gaussian sea can reach any height as there are no physical limits induced by wave breaking. Thus, the larger the area *A* or the time interval *D*, the greater the number of waves sampled in space-time, and unrealistically large amplitudes are likely to be sampled. Recently, Benetazzo et al.^59^ proposed an approach to limit the effects of the unboundedness of rare Gaussian space-time events. In particular, they bound the range of wave crest amplitudes of a given sea state in the finite interval $[0, h_{max}]$ as waves inevitably break and limit crest heights. The threshold $h_{max}=\xi _{max}H_s$ is chosen based on wave observations. For Draupner, Andrea and El Faro sea states Benetazzo et al.^59^ set $\xi _{max}=1.55$, but both Andrea and El Faro attained larger amplitudes (see Table 1). Such an approach was pioneered by Collins^45^ and Battjes^46^ in the early 70*s* (see Thornton and Guza^47^ for a comprehensive review). Hereafter, we refer to it as the Collins-Battjes method. In simple words, it relies on defining a sea state of *N* waves whose sample space consists of $N P_{max}$ non-breaking waves and $N(1-P_{max})$ breaking waves. Here, $P_{max}=Pr(h<h_{max})$ is the probability, or fraction of waves that do not break. All the mass probability of breaking waves is concentrated at $h=h_{max}$ and the associated probability density function (pdf) of crest heights has a Dirac delta spike $\delta (h-h_{max})$ of mass $(1-P_{max})$ at $h_{max}$ so that the probability of the entire sample space is 1. The pdfs reported by Benetazzo et al.^59^ in their Eqs. (13) and (16) miss the Dirac delta spike to concentrate the probability mass $(1-P_{max})$ at $h_{max}$. Following Collins-Battjes method^45–47^, we bound the Tayfun-Fedele exceedance probability in Eq. (4) as

8 $$\begin{aligned} P_{\mathrm {ST,bound}}(\xi ;A,D)=\left\{ \begin{array}{c} P_{\mathrm {ST}}(\xi ;A,D),\quad \;\xi \le \xi _{max},\\ \\ 0,\quad \quad \quad \qquad \xi >\xi _{max}. \end{array}\right. \end{aligned}$$

The correct pdf must have a Dirac delta spike at $\xi =\xi _{max}$ as

9 $$\begin{aligned} p_{\mathrm {ST,bound}}(\xi ;A,D)=\left\{ \begin{array}{c} -\frac{dP_{\mathrm {ST,bound}}}{d\xi },\;\quad \quad \qquad \quad \xi \le \xi _{max},\\ \\ (1-P_{max})\delta (\xi -\xi _{max}),\quad \xi =\xi _{max},\\ \\ 0,\;\;\quad \quad \quad \qquad \qquad \quad \quad \xi >\xi _{max}, \end{array}\right. \end{aligned}$$

where $P_{max}=Pr(h<h_{max})=1-P_{\mathrm {ST,bound}}(\xi _{max};A,D)$ and the probability of the entire sample space adds up to 1. The space-time probability of exceedance can be estimated by the associated Gumbel model^15^

10 $$\begin{aligned} P_{Gumbel}(\xi )=1-\exp \left[ -\exp \left( -\beta (\xi -\xi _{\mathrm {m}} \right) \right] ,\qquad 0\le \xi \le \xi _{max}, \end{aligned}$$

limited in the range $[0,\xi _{max}]$. The expected maximum surface height follows by integrating the exceedance probability over the finite range

11 $$\begin{aligned} \xi _{\mathrm {ST}}=\overline{h}_{\mathrm {ST}}/H_{s}=\int _{0}^{\xi _{max}} P_{\mathrm {ST,bound}}(\xi ;A,D)\,d\xi . \end{aligned}$$

The nonlinear mean maximum surface or crest height $h_{\mathrm {T}}$ expected at a point during the time interval *D* follows from Eq. (3) by setting $M_2=M_3=0$ and $M_1=N_{\mathrm {D}}$. We note in passing that for Gaia and Jan 2017 storms the unbounded TF model well describes the statistics of large crests at a point (see Figs. 6 and 7), and so for El-Faro^13^, Andrea and Draupner^12^ rogue waves. The second-order form of the ST model above ($\Lambda =0$) has been implemented in WAVEWATCH III^60^. The linear limit follows from Eq. (3) by setting $\mu =0$ and $\Lambda =0$.

To characterize rare space-time occurrences in third-order nonlinear random seas we consider the threshold $h_q=\xi _q H_s$ exceeded with probability *q* by the maximum surface height $\eta _{\max }$ over an area *A* during a sea state of duration *D*. This satisfies

12 $$\begin{aligned} P_{\mathrm {ST},bound}(\xi _q;A,D)=q. \end{aligned}$$

**Figure 9:**
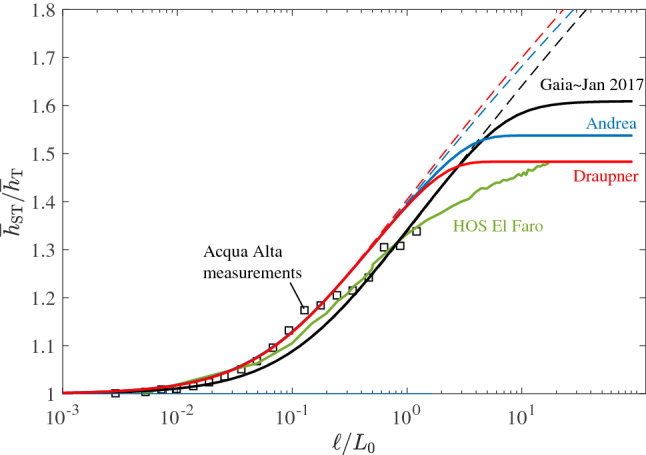
Space-time extremes: theoretical ratio $\overline{h}_{\mathrm {ST}}/\overline{h}_{\mathrm {T}}$ as a function of the area width $\ell /L_0$ for El Faro, Draupner and Andrea rogue sea states as well as the sea states at the peaks of Gaia and Jan 2017 storms. $\overline{h}_{\mathrm {ST}}$ is the mean maximum surface height expected over the area $\ell ^2$ during a sea state of duration $D=1$ hours and $\overline{h}_{\mathrm {T}}$ is the mean maximum surface height expected at a point. For comparisons, the empirical ST ratio from the El Faro HOS simulations (green solid line) together with the experimental observations at the Acqua Alta tower (squares) are also shown^16^. $L_0$ is the mean wavelength. Dashed lines denote the unbounded Gaussian predictions, whereas solid lines denote the bounded predictions using Collins-Battjes method^45–47^ with $\xi _{max}=1.55$.

In simple words, the maximum surface height $\eta _{\max }$ observed within the area *A* during *D* exceeds the threshold $h_{q}$ only in $q N$ realizations of an ensemble of *N* identical sea states. Consider the ST ratio $\overline{h}_{\mathrm {ST}}/\overline{h}_{\mathrm {T}}$, where $\overline{h}_{\mathrm {ST}}$ is the mean maximum surface height expected over the area $\ell ^2$ during a sea state of duration $D=1$ hour and $\overline{h}_{\mathrm {T}}$ is the mean maximum surface height expected at a point during the same time interval *D*. Figure 9 displays the unbounded ST ratios (dashed lines) as a function of the area width $\ell /L_0$ for the El Faro, Draupner and Andrea sea states^13^ as well as the sea states at the peaks of Gaia and Jan 2017 storms. We also depict the bounded ratios (solid lines) using Collins-Battjes method and the threshold $\xi _{max}=1.55$ as in Benetazzo et al.^59^. The ST ratios for Draupner and Andrea are estimated using the European Reanalysis (ERA)-interim data^52^. For comparisons, we also report the empirical ST ratio from the HOS-simulated El Faro sea state^13^ and the experimental observations at the Acqua Alta tower^16^. Fedele et al.^13^ noted that the unbounded predictions fairly agree with the El Faro HOS simulations and observations for small areas ($\ell \le L_0$), but they overestimate over larger areas. Note that Gaia and Jan 2017 give similar predictions and slightly smaller than the other sea states. As expected, the bounded predictions limit the growth of the statistical ensemble of waves in space-time. In particular, the maximum surface height expected over an area is bounded by the threshold $h_{max}=1.55H_s$, and it does not exceed 1.6 times the surface height expected at a point.

### Prediction of potential hazards for vessel navigation

Consider a vessel that navigates through a wave field of surface elevations $\eta (x,y,t)$ at a constant speed *V* along a straight path at an angle $\beta$ with respect to the *x* axis of a fixed Cartesian frame (*x*, *y*, *t*) over a time interval *D*. Define the occurrence frequency, or exceedance probability $P_e(h)$ that a rogue wave exceeding a crest height *h* is encountered by the vessel during its navigation path. $P_e(h)$ can be estimated as the probability that the maximum surface height exceeds *h* over the space-time volume $V_e$ spanned by the vessel in motion. To do so, we draw on Fedele et al.^13^ and consider the encountered wave field $\eta _c$ as seen by an observer in the frame $(x_e,y_e,t)$ moving with the vessel, where

13 $$\begin{aligned} x=x_e+V\cos (\beta )t,\quad y=y_e+V\sin (\beta )t, \end{aligned}$$

and (*x*, *y*, *t*) is the fixed Cartesian frame. The wave field encountered by the cruising vessel, or equivalently the field of surface fluctuations measured from the moving frame $(x_e,y_e,t)$ is

14 $$\begin{aligned} \eta _c(x_e,y_e,t)=\eta (x_e+V\cos (\beta )t,y_e+V\sin (\beta )t,t). \end{aligned}$$

For an observer in the fixed frame (*x*, *y*, *t*), $\eta$ is a random wave field homogeneous in space and stationary in time, and so is the encountered field $\eta _c$ as seen from an observer on the vessel in the moving frame $(x_e,y_e,t)$. However, $\eta _c$ will be non-stationary for a generic navigation route and variable vessel’s speed. The associated space-time covariance is given by

15 $$\begin{aligned} \Psi _c(X,Y,T)=\overline{\eta _c(x_e,y_e,t) \eta _c(x_e+X,y_e+Y,t+T)}=\int S(f,\theta )\cos (k_{x}X+k_{y}Y-2\pi f_{e}T) \mathrm {d}f\mathrm {d}\theta , \end{aligned}$$

where $S(f,\theta )$ is the directional wave spectrum of the sea state, $k_x=k\cos \theta$ and $k_y=k\sin \theta$, where *k* is the wavenumber related to the frequency *f* via the linear dispersion relation. The Doppler-shifted encountered, or apparent frequency is^61–63^

16 $$\begin{aligned} f_{e}=f-k V\cos (\theta -\beta )/(2\pi ). \end{aligned}$$

When the vessel moves faster than the waves coming from a direction $\theta$, the apparent frequency $f_{e}<0$ and waves appear to move away from an observer on the vessel. In this case, the direction of those waves should be reversed^61^, i.e. $\theta =\theta +\pi$, and $f_e$ set as positive. The spectral moments $m_{ijk}^{(e)}$ of the encountered random field $\eta _c$ are given by^13^

17 $$\begin{aligned} m_{ijk}^{e}=\frac{\partial ^{i+j+k}\Psi _c}{\partial X^i \partial Y^j \partial T^k}\Big |_{X=Y=T=0}=\int S(f,\theta )k_{x}^{i}k_{y}^{j}f_{e}^{k}\mathrm {d}f\mathrm {d}\theta . \end{aligned}$$

In the moving frame $(x_e,y_e,t)$, the vessel is stationary. As a consequence, the vessel spans the same area equal to its size *L* x *W* m$^2$ over the duration *D*, i.e. it covers the space-time volume $V_e=L W$ m$^2$ x *D* min. Here, *L* and *W* are the length and width of the vessel, respectively. The probability $P_e(h)$ that the maximum surface height over $V_e$ exceeds the threshold *h* can be estimated from the spectral moments $m_{ijk}^{e}$ of the encountered wave field using the space-time model^13, 15, 52^ in Eq. (3). In our analysis, we consider the worst-case scenario of the vessel cruising against the dominant wave direction of the sea state. In such conditions the moving vessel encounters more waves than if it were anchored at a fixed point because the apparent frequency of the encountered waves increases.

**Figure 10:**
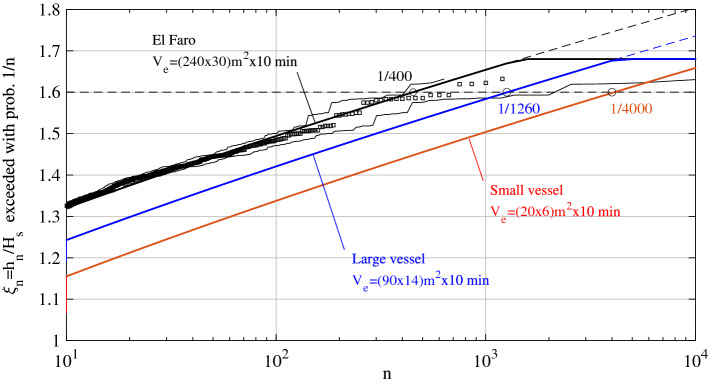
Theoretical bounded predictions (Collins-Battjes method^45–47^, $\xi _{max}=1.68$) for the maximum surface height $h_n/H_s$ exceeded with probability 1/*n* over the space-time volume $V_e=L W$ m$^2$ x *D* min spanned by the El Faro vessel (black lines), the Super Dvora Mk III-class patrol boat (red lines, small vessel) and the Sa’ar 6-class corvette (blue lines, large vessel). The vessels navigate against the dominant waves in the peak sea state of storm Gaia for a duration *D*. *L* and *W* are the length and width of the vessel. The numerical HOS predictions (squares) for El Faro are also reported^13^. Dashed lines denote the unbounded Gaussian predictions. Dashed horizontal line denotes the $1.6 H_s$ threshold.

As an application, we hypothesize the scenarios of two vessels of the Israeli Navy fleet navigating at their cruise speeds against the dominant waves of the peak sea state of Gaia over a time interval of 10 min. We consider the Super Dvora Mk III-class patrol boat, a vessel of small size (length 20 m and width 6 m) cruising at the average speed of  50 km/h (27 knots) and maximum speed of 93 km/h (50 knots). We also consider the Sa’ar 6-class corvette, a large vessel (length 90 m and width 14 m) cruising at the average speed of 30 km/h (17 knots) and maximum speed of 50 km/h (27 knots). Figure 10 depicts the bounded predictions (solid lines) of the nonlinear threshold $h_n/H_s$ exceeded with probability 1/*n* by the maximum surface height seen by the observer on the two vessels over the duration $D=10$ min. We used the Collins-Battjes method^45–47^ described in the preceding section and adopt $\xi _{max}=1.68$, which is the crest height of the largest simulated wave of the El Faro sea state^13^. The unbounded Gaussian predictions are also reported (dashed lines). Consider an observer on the large vessel navigating against the dominant waves of the peak sea state of Gaia at the average cruise speed of 30 km/h. The observer sees the vessel as stationary and the spanned space-time volume is $V_e=90$ x 14 $\text{m}^2$ x10 min. The probability that the maximum surface height exceeds $h=1.6H_s\approx 9$ m (here, $H_s=6.16$ m, see Table 1) when the vessel cruises along the dominant direction of waves is close to $P_e=1/1260$, as shown in Fig. 10. An observer on the small vessel navigating against the dominant waves at the average cruise speed of 50 km/h has a smaller probability $P_e=1/4000$ to encounter the rogue wave, because the vessel’s size is smaller than that of the large vessel. If the two vessels navigate along with the dominant waves $P_e$ reduces to 1/2400 (large vessel) and 1/10,500 (small vessel), because they will encounter less waves as the apparent wave frequency reduces.

For an observer fixed at a point of the sea, $P_e$ reduces to $1/10^5$ (see Fig. 8). We note in passing that if the two vessels navigate at their maximum speeds against the dominant waves, $P_e$ increases to 1/3160 (small vessel) and 1/1000 (large vessel).

Figure 10 also depicts the predictions for the El Faro vessel during Hurricane Joaquin based on HOS simulations^13^. The data suggests that the El Faro vessel was drifting at an average speed of approximately 2.5 m/s prior to its sinking^13^. Over a time interval of $D=10$ min prior sinking the probability to encounter a rogue wave that exceeds the threshold $h=1.6H_s\approx 14$ m is $P_e\approx 1/400$, in agreement with the predictions by Fedele et al.^13^. Thus, we find the same probabilities for the hypothetical scenario of the El Faro vessel drifting through the sea state at the storm peak of Gaia. However, in this case the rogue wave would exceed a smaller height, i.e. $1.6H_s\approx 9$ m since the significant wave height at the peak of Gaia ($H_s=6.16$ m) is smaller than that of the El Faro sea state ($H_s=9$ m, see Table 1).

## Conclusions

The Eastern Mediterranean is a basin of increasing interest being a climatology hot-spot and a natural treasures area, both ecologically and economically^1^. However, there is a lack of studies on the metocean characteristics of its extreme sea states and large waves. Such studies are of paramount importance, especially in light of global warming where weather events are likely to become more severe and more hazardous to offshore and coastal structures as well as to vessel navigation.

To fill the gap of knowledge on metocean properties of the Eastern Mediterrenean sea storms, we have presented a scientific investigation and prediction of wave extremes of that area. Our statistical analysis indicates that the largest observed waves during the two major storms recorded in the last five years have characteristics quite similar to those displayed by the El Faro, Andrea, and Draupner rogue waves^12, 13^. In particular, linear dispersive focusing enhanced by second order bound nonlinearities are the dominant factor in the generation of large waves. Third-order resonances and NLS-type modulational instabilities are ineffective in shaping large waves^12, 19^. Finally, we have carried out a novel space-time analysis of the most intense sea states and demonstrated the ability to predict potential rogue hazards for ships of various sizes and cruise speed navigating within the rough waters of the analyzed storms.

## Methods

### Buoy measurements

The ‘3 metre’ buoy manufactured by AXYS Technologies is located on the south part of the Exclusive Economic Zone of Israel. The buoy is moored at a depth of 260 m at the coordinates ($31^o$ $45'$ $41''$ Lat., $34^o$ $20'$ $30''$ Long.). This buoy operates by recording 18 minuets of heave displacements at the beginning of each hour with sampling frequency of $f_s=7.14$ Hz. Additional outputs are also elaborated by the internal buoy’s software, i.e. the directional energy density spectrum spectrum $S(\omega ,\theta )$ with a resolution of 3° covering 360°, and typical wave parameters such as the significant wave height, wave period and steepness. The same wave parameters were estimated independently from the the time series of the surface wave fluctuations provided by the buoy, and they were practically identical to the buoy’s outputs. For a cross-site validation, the buoy’s wave parameters have been compared to data acquired by the nearby onshore (i) Ashkelon port meteorological station^64^ and (ii) the DATAWELL Wave-Rider MKIII buoy^65^ 40-km away from our buoy, and moored 2 km offshore at a 24-m depth. In both cases, the buoy’s wave parameters showed similar trends to those acquired by the two instruments, while differing slightly in values. This is expected in light of the distance between the instruments’ locations and the physical characteristics of shallow waters and onshore environments.

### Wave parameters

The significant wave height $H_s$ is defined as the mean value $H_{1/3}$ of the highest one-third of wave heights. It can be estimated either from a zero-crossing analysis or more easily but approximately from the wave omnidirectional spectrum $S_o(f)=\int _{0}^{2\pi } S(f,\theta )\mathrm {d}{\theta }$ as $H_s \approx 4\sigma$, where $\sigma =\sqrt{m_0}$ is the standard deviation of surface elevations, $m_j=\int S_o(f) f^j\mathrm {d}f$ are spectral moments. Further, $S(f,\theta )$ is the directional wave spectrum with $\theta$ as the direction of waves at frequency *f*, and the cyclic frequency is $\omega =2\pi f$. In this paper, we use the spectral-based estimate, which according to our wave measurements is $5\%-10\%$ larger than $H_{1/3}$ estimated from the measured time series.

The dominant wave period $T_{p}=2\pi /\omega _p$ refers to the cyclic frequency $\omega _p$ of the spectral peak. The mean zero-crossing wave period $T_{0}$ is equal to $2\pi /\omega _0$, with $\omega _0=\sqrt{m_2/m_0}$. The associated wavelength $L_{0}=2\pi /k_0$ follows from the linear dispersion relation $\omega _0 = \sqrt{gk_0 \tanh (k_0 d)}$, with *d* the water depth. The mean spectral frequency is defined as $\omega _{m}=m_{1}/m_{0}$^23^ and the associated mean period $T_m$ is equal to $2\pi /\omega _m$. A characteristic wave steepness is defined as $\mu _m=k_m\sigma$, where $k_{m}$ is the wavenumber corresponding to the mean spectral frequency $\omega _{m}$^23^. The following quantitites are also introduced: $q_m = k_m d, Q_m = \tanh q_m$, the phase velocity $c_m = \omega _m/k_m$, the group velocity $c_g=c_m\left[ 1+2q_{m}/\mathrm {sinh(2}q_{m})\right] /2$.

The spectral bandwidth $\nu =(m_0 m_2/m_1^2-1)^{1/2}$ gives a measure of the frequency spreading. The angular spreading $\sigma _{\theta }=\sqrt{\int _0^{2\pi }D(\theta )(\theta -\theta _m)^2 \mathrm {d}\theta }$, where $D(\theta )=\int _0^{\infty }S(\omega ,\theta )\mathrm {d}\omega /\sigma ^2$ and $\theta _m=\int _0^{2\pi }D(\theta )\theta \mathrm {d}\theta$ is the mean direction. Note that $\omega _0=\omega _m\sqrt{1+\nu ^2}$. An alternative measure of spectral bandwidth is given by the Boccotti parameter $\psi ^{*}=\psi (\tau ^{*})$, which is the absolute value of the first minimum of the normalized covariance function $\psi (\tau )=\overline{\eta (t)\eta (t+\tau )}/\sigma ^{2}$ of the zero-mean surface displacement $\eta (t)$, which is attained at $\tau =\tau ^{*}$^66^ and $\ddot{\psi }^{*}=\ddot{\psi }(\tau ^{*})$ the corresponding second derivative with respect to $\tau$^66^.

The wave skewness $\lambda _3$ and the excess kurtosis $\lambda _{40}$ of the zero-mean surface elevation $\eta (t)$ are given by

18 $$\begin{aligned} \lambda _3=\overline{\eta ^3}/\sigma ^3,\qquad \lambda _{40}=\overline{\eta ^4}/\sigma ^4-3 \,. \end{aligned}$$

Here, overbars imply statistical averages and $\sigma$ is the standard deviation of surface wave elevations. Note that $\lambda _3$ was first derived by^67^. For second-order waves in deep water^25^

19 $$\begin{aligned} \lambda _{3}\approx 3\mu _m(1-\nu +\nu ^2), \end{aligned}$$

and^18^

20 $$\begin{aligned} 3\mu _m(1-\sqrt{2}\nu +\nu ^2) \le \lambda _3 \le 3\mu _m. \end{aligned}$$

Here, $\nu$ is the spectral bandwidth defined above and the characteristic wave steepness $\mu _m=k_m\sigma$, where $k_{m}$ is the wavenumber corresponding to the mean spectral frequency $\omega _{m}$^23^. For narrow-band waves, $\nu$ tends to zero and the associated skewness $\lambda _{3,NB}=3\mu _m$^23–25^. The excess kurtosis of weakly nonlinear random seas

21 $$\begin{aligned} \lambda _{40}=\lambda _{40}^{d}+\lambda _{40}^{b} \end{aligned}$$

comprises a dynamic component $\lambda _{40}^{d}$ due to nonlinear quasi-resonant wave-wave interactions^17, 26^ and a Stokes bound harmonic contribution $\lambda _{40}^{b}$^20^. In deep water it reduces to the simple form $\lambda _{40,NB}^{b}=18\mu _m^{2}=2\lambda _{3,NB}^2$^20, 26, 68^ where $\lambda _{3,NB}$ is the skewness of narrowband waves^23^. In deep water, the dynamic component $\lambda _{40}^{d}$ is given in terms of a six-fold integral^17, 19^ that depends on the Benjamin-Feir index $BFI=\sqrt{2}\mu _m/\nu$ and the parameter $R=\sigma _{\theta }^{2}/2\nu ^{2}$, which is a dimensionless measure of the multidirectionality of dominant waves^19, 26, 69^.

### The Tayfun-Fedele model for crest heights

The probability $P(\xi )$ that a wave crest observed at a fixed point of the ocean in time exceeds the threshold $\xi H_s$ can be described by the third-order Tayfun-Fedele model^24^,

22 $$\begin{aligned} P_{TF}(\xi )=\mathrm {Pr}\left[ h>\xi \,H_s\right] =\mathrm {exp}\left( -8\,\xi _{0}^{2}\right) \left[ 1+\varLambda \xi _{0}^{2}\left( 4\,\xi _{0}^{2}-1\right) \right] , \end{aligned}$$

where $\xi _{0}$ follows from the quadratic equation $\xi =\xi _{0}+2\mu \,\xi _{0}^{2}$^23^. Here, the Tayfun wave steepness $\mu =\lambda _{3}/3$ is of $O(\mu _m)$ and it is a measure of second-order bound nonlinearities as it relates to the skewness $\lambda _{3}$ of surface elevations^25^. The parameter $\varLambda =\lambda _{40}+2\lambda _{22}+\lambda _{04}$ is a measure of third-order nonlinearities and is a function of the fourth order cumulants $\lambda _{nm}$ of the water surface elevation $\eta$ and its Hilbert transform $\hat{\eta }$^24^. In particular, $\lambda _{22}=\overline{\eta ^2\hat{\eta }^2}/\sigma ^4-1$ and $\lambda _{04}=\overline{\hat{\eta }^4}/\sigma ^4-3$. In our studies $\varLambda$ is approximated solely in terms of the excess kurtosis as $\varLambda _{\mathrm {appr}}={8\lambda _{40}}/{3}$ by assuming the relations between cumulants^70^ $\lambda _{22}=\lambda _{40}/3$ and $\lambda _{04}=\lambda _{40}$. These, to date, have been proven to hold for linear and second-order narrowband waves only^27^. For third-order nonlinear seas, our numerical studies indicate that $\varLambda \approx \varLambda _{\mathrm {appr}}$ within a $3\%$ relative error in agreement with observations^71, 72^.

For second-order seas, referred to as Tayfun sea states^73^, $\varLambda =0$ only and $P_{TF}$ in Eq. (22) yields the Tayfun (T) distribution^23^

23 $$\begin{aligned} P_{T}(\xi )=\mathrm {exp}\left( -8{\xi _{0}^2}\right) . \end{aligned}$$

For Gaussian seas, $\mu =0$ and $\varLambda =0$ and $P_{TF}$ reduces to the Rayleigh (R) distribution

24 $$\begin{aligned} P_{R}(\xi )=\mathrm {exp}\left( -8{\xi ^{2}}\right) . \end{aligned}$$

Fedele and Tayfun^25^ proved that the Tayfun distribution represents an exact result for large second order wave crest heights and it depends solely on the steepness parameter defined as $\mu =\lambda _{3}/3$.

### The modified narrowband (MNB) model

Tayfun and Alkhalidi^41^ introduced the MNB model as an improvement of the conventional weakly-nonlinear second-order finite-depth narrowband model so as to avoid certain unrealistic features of that model in relatively shallow waters and to extend its applicability to highly nonlinear waves propagating from deep water to the shoaling and surf zones. The MNB exceedance probability for crest heights is given by

25 $$\begin{aligned} P_{MNB}(\xi )=\mathrm {Pr}\left[ h>\xi \,H_s\right] =\mathrm {exp}\left( -8{\xi _{0}^2}\right) , \end{aligned}$$

where $\xi _{0}$ follows from the quadratic equation^11, 41^

$$\begin{aligned} \xi =\left( \xi _{0}+2\varepsilon \xi _{0}^{2}\right) /\alpha _1, \end{aligned}$$

where

$$\begin{aligned} \varepsilon &=0.3571\lambda _{3}-0.0227\lambda _{3}^{2}+0.0444\lambda _{3}^{3},\\ \alpha _1&=1+0.0146\lambda _3+0.0147\lambda _3^2+0.0219\lambda _3^3, \end{aligned}$$

and the associated excess kurtosis

$$\begin{aligned} \lambda _{40,MNB}=0.0276\lambda _{3}+1.4275\lambda _{3}^{2}-0.0063\lambda _{3}^{3}. \end{aligned}$$

The MNB model is valid over the range of skewness values $0\le \lambda _3\le 2$.

### The Forristall model

The exceedance probability is given by^43^

26 $$\begin{aligned} P_{F}(\xi )=\mathrm {exp}\left( -{(\xi /\alpha )^{\beta }}\right) , \end{aligned}$$

where $\alpha =0.3536+0.2561 S_1+0.0800 U_r$, $\beta =2-1.7912 S_1-0.5302 U_r+0.284 U_r^2$ for multi-directional (short-crested) seas. Here, $S_1=2\pi H_s/(g T_m^2)$ is a characteristic wave steepness and the Ursell number $U_r=H_s/(k_m^2 d^3)$, where $k_m$ is the wavenumber associated with the mean period $T_m=m_0/m_1$ and *d* is the water depth.

### The generalized Boccotti and Tayfun models for crest-to-trough (wave) heights

The third-order nonlinear statistics for crest-to-trough wave heights is described in terms of the generalized Boccotti distribution^42, 66^.

27 $$\begin{aligned} P_{B}(y)=\mathrm {Pr}\left[ H>y\,H_s\right] =\frac{1+\ddot{\psi }^{*}}{\sqrt{2\,\ddot{\psi }^{*}\left( 1+\psi ^{*}\right) }}\mathrm {exp} \left( -\frac{4\,y^{2}}{1+\psi ^{*}}\right) \left[ 1+\frac{\varLambda \,y^{2}}{1+\psi ^{*}}\left( \frac{y^{2}}{1+\psi ^{*}}-\frac{1}{2}\right) \right], \end{aligned}$$

where the Boccotti parameter $\psi ^*$ and $\ddot{\psi }^{*}$ are defined above in the section where statistical parameters are described. For Gaussian seas ($\varLambda =0$), the original Boccotti model^66^ is recovered

28 $$\begin{aligned} P_{B}(y)=\mathrm {Pr}\left[ H>y\,H_s\right] =\frac{1+\ddot{\psi }^{*}}{\sqrt{2\,\ddot{\psi }^{*}\left( 1+\psi ^{*}\right) }}\mathrm {exp} \left( -\frac{4\,y^{2}}{1+\psi ^{*}}\right) , \end{aligned}$$

The Tayfun model for wave heights is given by^24, 44^

29 $$\begin{aligned} P_{T}(y)=\mathrm {Pr}\left[ H>y\,H_s\right] =\sqrt{\frac{1+r_m}{2 r_m}}\left( 1+\frac{1-r_m^2}{64 r_m y^2}\right) \mathrm {exp}\left( -\frac{4\,y^{2}}{1+r_m}\right) , \end{aligned}$$

where $r_m=r(T_m/2)$ is the value of the envelope *r*(*t*) of the covariance $\psi (t)$ at $t=T_m/2$.

### Space-time statistical parameters

For space-time extremes, the coefficients in Eq. (3) are given by^15, 48^

$$\begin{aligned} M_{3}= & {} 2\pi \frac{D}{\overline{T}}\frac{\ell _{x}}{\overline{L_{x}}}\frac{\ell _{y}}{\overline{L_{y}}}\alpha _{xyt},\\ M_{2}= & {} \sqrt{2\pi }\left( \frac{D}{\overline{T}}\frac{\ell _{x}}{\overline{L_{x}}}\sqrt{1-\alpha _{xt}^{2}}+\frac{D}{\overline{T}}\frac{\ell _{y}}{\overline{L_{y}}}\sqrt{1-\alpha _{yt}^{2}}+\frac{\ell _{x}}{\overline{L_{x}}}\frac{\ell _{y}}{\overline{L_{y}}}\sqrt{1-\alpha _{xy}^{2}}\right) ,\\ M_{1}= & {} N_{D}+N_{x}+N_{y}, \end{aligned}$$

where

$$\begin{aligned} N_{D}=\frac{D}{\overline{T}},\qquad N_{x}=\frac{\ell _{x}}{\overline{L_{x}}},\qquad N_{y}=\frac{\ell _{y}}{\overline{L_{y}}} \end{aligned}$$

are the average number of waves occurring during the time interval D and along the x and y sides of length $\ell _{x}$ and $\ell _{y}$ respectively. They all depend on the mean period $\overline{T}$, mean wavelengths $\overline{L_{x}}$ and $\overline{L_{y}}$ in *x* and *y* directions:

$$\begin{aligned} \overline{T}=2\pi \sqrt{\frac{m_{000}}{m_{002}}},\qquad \overline{L_{x}} =2\pi \sqrt{\frac{m_{000}}{m_{200}}},\qquad \overline{L_{y}}=2\pi \sqrt{\frac{m_{000}}{m_{020}}} \end{aligned}$$

and

$$\begin{aligned} \alpha _{xyt}=\sqrt{1-\alpha _{xt}^{2}-\alpha _{yt}^{2}- \alpha _{xy}^{2}+2\alpha _{xt}\alpha _{yt}\alpha _{xy}}. \end{aligned}$$

Here,

$$\begin{aligned} m_{ijk}=\iint k_{x}^{i}k_{y}^{j}f^{k}S(f,\theta )dfd\theta \end{aligned}$$

are the moments of the directional spectrum $S(f,\theta )$ and

$$\begin{aligned} \alpha _{xt}=\frac{m_{101}}{\sqrt{m_{200}m_{002}}},\qquad \alpha _{yt}=\frac{m_{011}}{\sqrt{m_{020}m_{002}}},\qquad \alpha _{xy}=\frac{m_{110}}{\sqrt{m_{200}m_{020}}}. \end{aligned}$$

## Data Availability

All the publicly available data for El-Faro are posted on the National Transportation Safety Board (NTSB) website (https://www.ntsb.gov).

## References

[1] Flaounas E (2022). Mediterranean cyclones: Current knowledge and open questions on dynamics, prediction, climatology and impacts. Weather Clim. Dyn..

[2] Dysthe KB, Krogstad HE, Muller P (2008). Oceanic rogue waves. Ann. Rev. Fluid Mech..

[3] Sunde, A. Kjempebølger i nordsjøen (extreme waves in the north sea). Vær & Klima (1995).

[4] Haver, S. A possible freak wave event measured at the Draupner Jacket January 1 1995. In *Rogue Waves*, Vol. 2004, 1–8 (2004).

[5] Karin Magnusson A, Donelan MA (2013). The Andrea wave characteristics of a measured north sea rogue wave. J. Offshore Mech. Arctic Eng..

[6] Gemmrich J, Cicon L (2022). Generation mechanism and prediction of an observed extreme rogue wave. Sci. Rep..

[7] Cavaleri, L. & Bertotti, L. The voyager storm in the mediterranean sea. In *Proceedings of the 10th International Workshop on Wave Hindcasting and Forecasting and Coastal Hazard Symposium, Oahu, HI, USA*, 11–16 (2007).

[8] Cavaleri L (2012). Rogue waves in crossing seas: The louis majesty accident. J. Geophys. Res. Oceans.

[9] Cavaleri L (2021). An exceptionally high wave at the CNR-ISMAR oceanographic tower in the Northern Adriatic Sea. Sci. Data.

[10] Knobler S, Bar D, Cohen R, Liberzon D (2021). Wave height distributions and rogue waves in the Eastern Mediterranean. J. Mar. Sci. Eng..

[11] Shani-Zerbib A, Tayfun MA, Liberzon D (2021). Statistics of fetch-limited wind waves observed along the western coast of the Gulf of Aqaba. Ocean Eng..

[12] Fedele F, Brennan J, Ponce de León S, Dudley J, Dias F (2016). Real world ocean rogue waves explained without the modulational instability. Sci. Rep..

[13] Fedele F, Lugni C, Chawla A (2017). The sinking of the el faro: predicting real world rogue waves during Hurricane Joaquin. Sci. Rep..

[14] Fedele F, Herterich J, Tayfun A, Dias F (2019). Large nearshore storm waves off the Irish coast. Sci. Rep..

[15] Fedele F (2012). Space-time extremes in short-crested storm seas. J. Phys. Oceanogr..

[16] Fedele F (2013). Space-time measurements of oceanic sea states. Ocean Model..

[17] Janssen PAEM (2003). Nonlinear four-wave interactions and freak waves. J. Phys. Oceanogr..

[18] Tayfun MA (2006). Statistics of nonlinear wave crests and groups. Ocean Eng..

[19] Fedele F (2015). On the kurtosis of ocean waves in deep water. J. Fluid Mech..

[20] Janssen PAEM (2009). On some consequences of the canonical transformation in the Hamiltonian theory of water waves. J. Fluid Mech..

[21] Toffoli A, Babanin A, Onorato M, Waseda T (2010). Maximum steepness of oceanic waves: Field and laboratory experiments. Geophys. Res. Lett..

[22] Barthelemy X (2018). On a unified breaking onset threshold for gravity waves in deep and intermediate depth water. J. Fluid Mech..

[23] Tayfun MA (1980). Narrow-band nonlinear sea waves. J. Geophys. Res. Oceans.

[24] Tayfun MA, Fedele F (2007). Wave-height distributions and nonlinear effects. Ocean Eng..

[25] Fedele F, Tayfun MA (2009). On nonlinear wave groups and crest statistics. J. Fluid Mech..

[26] Janssen, P. A. E. M. & Bidlot, J. R. On the extension of the freak wave warning system and its verification. Tech. Memo 588, ECMWF (2009).

[27] Tayfun MA, Lo J (1990). Nonlinear effects on wave envelope and phase. J. Waterway Port Coast. Ocean Eng..

[28] Fedele F (2008). Rogue waves in oceanic turbulence. Phys. D.

[29] Fedele F (2014). On certain properties of the compact Zakharov equation. J. Fluid Mech..

[30] Onorato M (2009). Statistical properties of mechanically generated surface gravity waves: A laboratory experiment in a three-dimensional wave basin. J. Fluid Mech..

[31] Waseda T, Kinoshita T, Tamura H (2009). Evolution of a random directional wave and freak wave occurrence. J. Phys. Oceanogr..

[32] Toffoli A (2010). Evolution of weakly nonlinear random directional waves: Laboratory experiments and numerical simulations. J. Fluid Mech..

[33] Annenkov SY, Shrira VI (2013). Large-time evolution of statistical moments of wind-wave fields. J. Fluid Mech..

[34] Annenkov SY, Shrira VI (2014). Evaluation of skewness and kurtosis of wind waves parameterized by JONSWAP spectra. J. Phys. Oceanogr..

[35] Tayfun MA (2008). Distributions of envelope and phase in wind waves. J. Phys. Oceanogr..

[36] Christou M, Ewans K (2014). Field measurements of rogue water waves. J. Phys. Oceanogr..

[37] Mei CC (1989). The Applied Dynamics of Water Waves.

[38] Fenton JD (1985). A fifth-order stokes theory for steady waves. J. Waterway Port Coast. Ocean Eng..

[39] Knobler S, Winiarska E, Babanin A, Liberzon D (2022). Wave breaking probabilities under wind forcing in open sea and laboratory. Phys. Fluids.

[40] Trulsen K (1998). Crest pairing predicted by modulation theory. J. Geophys. Res. Oceans.

[41] Tayfun MA, Alkhalidi M (2020). Distribution of sea-surface elevations in intermediate and shallow water depths. Coast. Eng..

[42] Alkhalidi MA, Tayfun MA (2013). Generalized Boccotti distribution for nonlinear wave heights. Ocean Eng..

[43] Forristall GZ (2000). Wave crest distributions: Observations and second-order theory. J. Phys. Oceanogr..

[44] Tayfun MA (1990). Distribution of large wave heights. J. Waterway Port Coast. Ocean Eng..

[45] Collins, J. I. Probabilities of breaking wave characteristics. In *Proceedings of the 13th International Conference Coastal Engineering*, 399–412 (American Society of Civil Engineers, 1970).

[46] Battjes, J. A. Set-up due to irregular waves. In *Proceedings of the 13th International Conference Coastal Engineering*, 1993–2004 (American Society of Civil Engineers, 1972).

[47] Thornton EB, Guza RT (1983). Transformation of wave height distribution. J. Geophys. Res. Oceans.

[48] Baxevani A, Rychlik I (2006). Maxima for Gaussian seas. Ocean Eng..

[49] Adler RJ (1981). The Geometry of Random Fields.

[50] Adler RJ, Taylor JE (2009). Random Fields and Geometry.

[51] Adler, R. J. On excursion sets, tube formulas and maxima of random fields. *Ann. Appl. Probab.* 1–74 (2000).

[52] Fedele, F. On oceanic rogue waves. arXiv preprint arXiv:1501.03370 (2015).

[53] Piterbarg, V. I. *Asymptotic Methods in the Theory of Gaussian Processes and Fields*, Vol. 148 (AMS ser. Translations of Mathematical Monographs, 1995).

[54] Socquet-Juglard H, Dysthe KB, Trulsen K, Krogstad HE, Liu J (2005). Probability distributions of surface gravity waves during spectral changes. J. Fluid Mech..

[55] Forristall, G. Z. Maximum crest heights under a model TLP deck. In *ASME 2011 30th International Conference on Ocean, Offshore and Arctic Engineering*, 571–577 (American Society of Mechanical Engineers, 2011).

[56] Forristall, G. Z. Maximum crest heights over an area: laboratory measurements compared to theory. In |textitASME 2015 34th International Conference on Ocean, Offshore and Arctic Engineering, OMAE2015–41061 (American Society of Mechanical Engineers, 2015).

[57] Cavaleri L (2016). The Draupner wave: A fresh look and the emerging view. J. Geophys. Res. Oceans.

[58] Fedele F, Chandre C, Farazmand M (2016). Kinematics of fluid particles on the sea surface: Hamiltonian theory. J. Fluid Mech..

[59] Benetazzo A, Barbariol F, Davison S (2020). Short-term/range extreme-value probability distributions of upper bounded space-time maximum ocean waves. J. Mar. Sci. Eng..

[60] Barbariol F, Pierson W (2017). Numerical modeling of space-time wave extremes using wavewatch III. Ocean Dyn..

[61] St. Denis M, Pierson W (1953). On the motions of ships in confused seas. Trans. Soc. Naval Archit. Mar. Eng..

[62] Lindgren G, Rychlik I, Prevosto M (1999). Stochastic doppler shift and encountered wave period distributions in Gaussian waves. Ocean Eng..

[63] Podgórski K, Rychlik I, Machado UE (2000). Exact distributions for apparent waves in irregular seas. Ocean Eng..

[64] Service, I. M. Asheqelon port meteorological station. https://ims.gov.il/en/node/209.

[65] BV, D. Wave-rider mkiii buoy. https://www.datawell.nl/.

[66] Boccotti P (2000). Wave Mechanics for Ocean Engineering.

[67] Marthinsen, T. & Winterstein, S. R. *On the Skewness of Random Surface Waves* 7 (1992).

[68] Janssen PAEM (2014). On a random time series analysis valid for arbitrary spectral shape. J. Fluid Mech..

[69] Mori N, Onorato M, Janssen PAEM (2011). On the estimation of the kurtosis in directional sea states for freak wave forecasting. J. Phys. Oceanogr..

[70] Mori N, Janssen PAEM (2006). On kurtosis and occurrence probability of freak waves. J. Phys. Oceanogr..

[71] Fedele F, Cherneva Z, Tayfun MA, Soares CG (2010). Nonlinear Schrödinger invariants and wave statistics. Phys. Fluids.

[72] Tayfun, M. A. & Fedele, F. Expected shape of extreme waves in storm seas. In *ASME 2007 26th International Conference on Offshore Mechanics and Arctic Engineering*, OMAE2007–29073 (American Society of Mechanical Engineers, 2007).

[73] Trulsen K, Nieto Borge JC, Gramstad O, Aouf L, Lefèvre J-M (2015). Crossing sea state and rogue wave probability during the Prestige accident. J. Geophys. Res. Oceans.

